# Can ABCF2 protein expression predict the prognosis of uterine cancer?

**DOI:** 10.1038/sj.bjc.6604734

**Published:** 2008-11-11

**Authors:** S Nishimura, H Tsuda, Y Miyagi, A Hirasawa, A Suzuki, F Kataoka, H Nomura, T Chiyoda, K Banno, T Fujii, N Susumu, D Aoki

**Affiliations:** 1 Department of Obstetrics and Gynecology, Osaka City General Hospital, Osaka, Japan; 2 Department of Obstetrics and Gynecology, Keio University School of Medicine, Tokyo Japan; 3 Department of Obstetrics and Gynecology, Okayama Ohfuku Clinic, Okayama, Japan

**Keywords:** ABC transporter, cervical cancer, endometrial cancer, prognosis

## Abstract

Uterine cervical and endometrial cancers are common malignant solid neoplasms for which there are no useful prognostic markers. In this study, we evaluate the relationship between ATP-binding cassette superfamily F2 (ABCF2) expression and clinical factors including clinical stage, histologic type, grade and prognosis in uterine cervical and endometrial cancer. Two hundred and sixty seven cervical and 103 endometrial cancers were studied. ATP-binding cassette superfamily F2 cytoplasmic expression was detected by immunohistochemical staining and scored as positive or negative. Among cervical cancer cases, 149 (55.8%) expressed ABCF2. The overall survival was longer in ABCF2-negative than ABCF2-positive cases (*P*=0.0069). Statistically significant prognostic factors for survival were ABCF2 positivity (risk ratio (rr)=1.437), old age (rr=1.550) and advanced stage (rr=2.577). ATP-binding cassette superfamily F2 positivity was an independent prognostic factor by multivariate proportional hazard test (*P*=0.0002). Among endometrial cancer cases, 72 (69.9%) were cytoplasmic ABCF2 positive. However, there was no significant relationship between ABCF2 expression and age, clinical stage, histologic type, histologic grade, oestrogen receptor status or prognosis. ATP-binding cassette superfamily F2 expression may be a useful prognostic marker in cervical but not endometrial cancer. The role of ABCF2 protein may differ depending on the type of cancer.

Carcinoma of the uterine cervix is a common malignant solid neoplasm in Japanese women and its incidence in young women is increasing (Ioka *et al*, 2003; The Japan Cancer Surveillance Research Group, in press). The prognosis is influenced by health condition such as anaemia, hypertension, neutrophil count or diabetes, clinical stage including tumour volume, endometrial extension and lymph node involvement (Stehman *et al*, 2000). Several studies have assessed the role of human papillomavirus (HPV) and HPV type on outcome with conflicting results (Randall *et al*, 2005). Proliferation, apoptosis and other cellular characteristics have been suggested to correlate with clinical outcome although these associations have not been conclusively established (Riou *et al*, 1988; Sagae *et al*, 1989; Koulos *et al*, 1993; Tsang *et al*, 1995; Gaffney *et al*, 2003). Several genes that control cell growth and differentiation have been investigated as prognostic markers in cervical cancer, but as yet no clearly useful marker has been identified.

Endometrial cancer is also increasing in Japan (Ioka *et al*, 2003; The Japan Cancer Surveillance Research Group, in press) and the prognosis is reported to be influenced by histologic type, grade and clinical stage (Trope *et al*, 2005). Mutations of p53 gene, HER-2/neu amplification and reduced E-cadherin expression have been reported to be poor prognostic indicators but their importance remains to be established (Hamel *et al*, 1996; Lundgren *et al*, 2002; Moreno-Bueno *et al*, 2002). New biomarkers able to predict the prognosis of uterine cervical and endometrial cancers are therefore urgently needed and would be extremely useful.

We have produced an antibody to the ATP-binding cassette superfamily F (ABCF2) protein and demonstrated that ABCF2 protein expression is higher in clear cell adenocarcinoma (CCC) of the ovary than in other histologic types and may predict chemotherapy response and prognosis (Tsuda *et al*, 2005a; Nishimura *et al*, 2007). ATP-binding cassette superfamily F2 is a member of the GCN20 subfamily of the ABC transporter superfamily (Vasquez de Aldana *et al*, 1995; Allikmets *et al*, 1996; Kerr, 2004). Yasui *et al* reported that the ABCF2 gene is amplified in a chemoresistant ovarian cancer cell line (t24/cDDp10), with chromosome gain at 7q34–36 (Yasui *et al*, 2005). In this study, we evaluate the relationship between ABCF2 expression and clinical stage, histologic type, histologic grade and prognosis in uterine cervical and endometrial cancer.

## Materials and methods

### Clinical samples

Between February 1994 and December 2004, 385 samples (267 invasive cervical cancers, 103 endometrial cancer, 5 proliferative endometrium, 5 secretary phase endometrium and 5 normal cervical epithelium) of paraffin-embedded tissues were collected at Osaka City General Hospital. All patient-derived paraffin sections were archived under the protocols approved by the institutional review board (IRB) of Osaka City General Hospital.

Of 267 invasive cervical cancers, 146 were stage I, 67 stage II, 33 stage III and 21 stage IV. Two hundred and twenty six were squamous cell carcinoma, 26 adenocarcinoma and 15 adenosquamous cell carcinoma. Among the 103 endometrial cancers, 63 were stage I, 6 stage II, 31 stage III and 3 stage IV. Eighty-six were endometrioid adenocarcinoma, six adenoachanthoma, five adenosquamous carcinoma, four CCC and two undifferentiated carcinoma. Forty-four were grade1, 42 grade 2 and 13 grade 3. The median ages of cervical and endometrial cancer patients were 53 (range 22–92) and 55 years (range 32–77), respectively. Histologic diagnosis was confirmed by microscopic examination of hematoxylin-and-eosin-stained sections according to the International Federation of Gynecology and Obstetrics (FIGO) system.

As primary therapy 70 cervical cancer patients received radiation therapy and 197 surgery. After primary therapy, 55 had adjuvant radiotherapy and 10 platinum based adjuvant chemotherapy. Twenty-nine patients received platinum-based neoadjuvant chemotherapy. All endometrial cancer patients were first treated surgerically. Post-operatively 51 received platinum-based adjuvant chemotherapy and 2 adjuvant radiotherapy.

### Immunohistochemistry

ATP-binding cassette superfamily F2 protein expression was detected immunohistochemically using a polyclonal anti-ABCF2 antibody generated by immunising rabbits with a purified full-length ABCF2 fusion protein. The specificity of the antibody was determined by western blot and immunohistochemical analyses on human embryonic kidney cells (293T) transfected with an expression vector pcDNA3.1 or with the vector containing either a full-length ABCF2 coding sequence or a full-length ABCF2 coding sequence with a myc and a His tag (Tsuda *et al*, 2005b).

Histological sections (4 *μ*m) were affixed to glass slides, dewaxed and rehydrated. The sections were incubated in 3% hydrogen peroxide for 10 min at room temperature to quench endogeneous peroxidase activity and incubated with the ABCF2 antibody (1 : 5000) at 4°C overnight. After rinsing, the sections were incubated for 30 min with rabbit EnVision+ Peroxidase (DAKO, Glostrup, Denmark). Peroxidase activity was visualised by applying diaminobenzidine chromogen containing 0.05% hydrogen peroxide for 2–10 min at room temperature, and the sections were counterstained with hematoxylin. Slides were read by two independent pathologists, who were blinded to the clinical background of the patients. Evaluation of ABCF2 positives were reported previously (Tsuda *et al*, 2005a; Nishimura *et al*, 2007). ATP-binding cassette superfamily F2 protein is a member of ABC transporter superfamily and the GCN20 subfamily (Vasquez de Aldana *et al*, 1995) and we demonstrated that ABCF2 protein is predominantly located in the cytoplasm of cells (Tsuda *et al*, 2005a; Nishimura *et al*, 2007). Thus, in this study, we judged the positivities of this protein based on the cytoplasmic staining into either positive or negative.

Slides from epithelial ovarian cancer patients confirmed to be ABCF2 positive or negative in previous reports that were used as controls (Tsuda *et al*, 2005a; Nishimura *et al*, 2007).

### Statistical analysis

The relationship between ABCF2 expression and age, clinical stage and histologic type were analysed by *χ*^2^ test. Patients were categorised as young or old (>53 years) and early or advanced (not less than stage IIIa) disease. Factors influencing 5-year survival were analysed by both Cox's proportional hazard test and the Kaplan–Meier test. After investigation of the multicollinearity of these factors, multivariate Cox's proportional hazard test was applied. A *P*-value of <0.05 was considered statistically significant.

## Results

### ABCF2 expression

**Figure 1:**
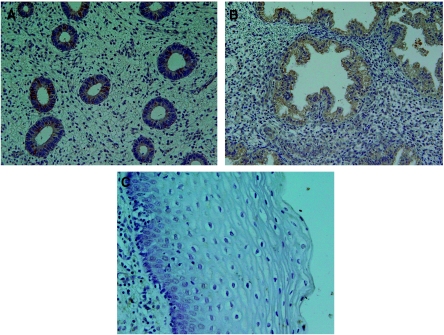
(**A**) ATP-binding cassette superfamily F2-positive proliferative endometrium. (**B**) ATP-binding cassette superfamily F2-positive secretary endometrium. (**C**) ATP-binding cassette superfamily F2-negative cervical epithelium.

**Figure 2:**
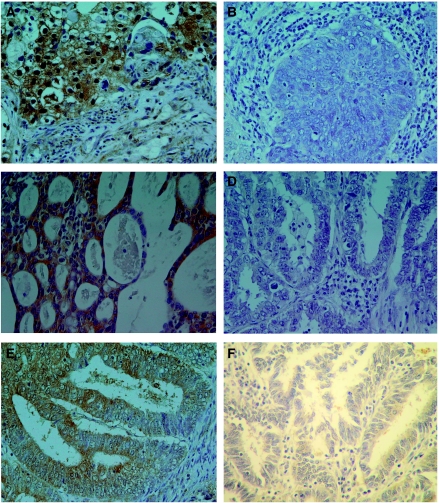
ATP-binding cassette superfamily F2 immunohistochemical staining in cervical and endometrial cancer. (**A**) ATP-binding cassette superfamily F2-positive cervical cancer (squamous cell carcinoma). (**B**) ATP-binding cassette superfamily F2-negative cervical cancer (squamous cell carcinoma). (**C**) ATP-binding cassette superfamily F2-positive cervical cancer (adenocarcinoma). (**D**) ATP-binding cassette superfamily F2-negative cervical cancer (adenocarcinoma). (**E**) ATP-binding cassette superfamily F2-positive endometrial cancer. (**F**) ATP-binding cassette superfamily F2-negative endometrial cancer.

In one of seven proliferative endometriums and six of eight secretary endometriums, ABCF2 cytoplasmic weakly immunostaining was detected; however, in normal cervical epithelium, no ABCF2 immunostaining was detected (Figure 1A–C). Among cervical cancer cases, 149 (55.8%) were cytoplasmic ABCF2 positive. ATP-binding cassette superfamily F2 was more frequently expressed in advanced (stage III+IV) than in early (stage I+II) cases (75.9 *vs* 50.7%, *P*=0.001). However, there was no significant relationship between age, histologic type and ABCF2 expression (Table 1). Among endometrial cancer cases, 72 (69.9%) were cytoplasmic ABCF2 positive. There was no association between age, clinical stage, histologic type, histologic grade, oestrogen receptor status and ABCF2 expression (Table 2). Representative ABCF2 staining is shown in Figure 2.

### Relationship between ABCF2 expression and overall survival (OS)

**Figure 3:**
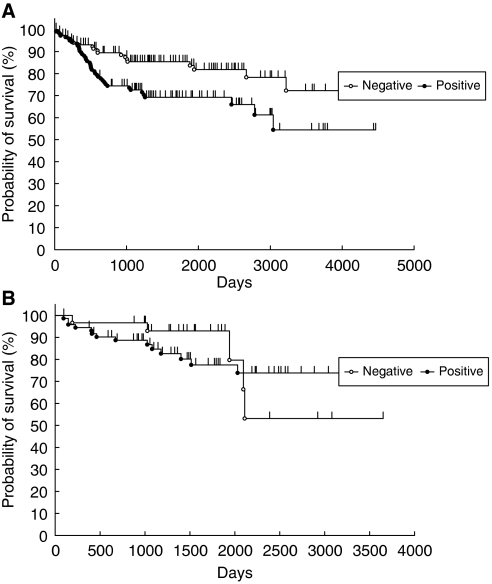
(**A**) Relationship between ABCF2 expression and OS in cervical cancer. ATP-binding cassette superfamily F2-negative cases show longer OS than in ABCF2-positive cases (*P*=0.0069). (**B**) Relationship between ABCF2 expression and the OS in endometrial cancer. ATP-binding cassette superfamily F2 expression and OS are not related (*P*=0.67).

The median follow-up period for cervical cancer patients was 1280 days (20–4679 days). Overall survival was significantly related to age and clinical stage but not to histologic type. The OS was longer in stage I+II than in stage III+IV cases (*P*<0.0001) and in younger than older cases (*P*=0.0007). Twenty of 118 ABCF2 negative and 41 of 149 ABCF2-positive cases died. Overall survival of ABCF2 negative was longer than ABCF2-positive cases (*P*=0.0069) (Figure 3A). Prognostic factors influencing survival were ABCF2 positivity (risk ratio (rr)=1.437; *P*_h_=0.0063 by proportional hazard test, *P*_l_=0.0069 by log-rank and *P*_w_=0.001 by Wilcoxon test), old age (rr=1.550; *P*_h_=0.0008, *P*_l_=0.0007 and *P*_w_=0.0015) and advanced stage (rr=2.577; *P*_h_<0.0001, *P*_l_<0.0001 and *P*_w_<0.0001) (Table 3). ATP-binding cassette superfamily F2 positivity and old age were independent significant prognostic factors by multivariate proportional hazard test (*P*=0.0002). The rrs for negative ABCF2 and old age were 0.720 (0.544–0.936; 95% CI, *P*=0.00137) and 1.510 (1.167–1.978, *P*=0.0016), respectively.

For endometrial cancer cases the median follow up period was 1429 days (93–3675 days). Overall survival was longer in stage I+II disease than in stage III+IV cases (*P*<0.0001). Endometrioid adenocarcinoma had better OS than non-endometrioid adenocarcinoma (*P*=0.001). However, there was no relationship between ABCF2 expression and OS (*P*=0.67) (Figure 3B).

## Discussion

Recently, we reported that ABCF2 expression was related to prognosis in ovarian CCC and breast cancer. In ovarian CCC, ABCF2 expression was associated with chemoresistance and OS (Tsuda *et al*, 2005a, 2005b; Ogawa *et al*, 2006), while in breast cancer, lack of ABCF2 expression was associated with increased disease-free survival (Ogawa *et al*, 2006). ATP-binding cassette superfamily F2 is a member of the GCN20 subfamily of the ABC transporter superfamily (Vasquez de Aldana *et al*, 1995). Similar to other members of the ABC family, ABCF2 contains a pair of nucleotide-binding domains but it lacks a transmembrane domain (Allikmets *et al*, 1996; Kerr, 2004), suggesting that it is unlikely to function as a cell membrane transporter, as do other members of the ABC family. ATP-binding cassette superfamily F2 may be involved in translational control, antibiotic resistance and RNase L inhibition (Trope *et al*, 2005). Although the ABCF2 gene is amplified in a chemoresistant ovarian cancer cell line (t24/cDDp10) with chromosome gain at 7q34–36 (Yasui *et al*, 2005), its role in tumour biology remains to be established. In this study, we examined the relationship between ABCF2 expression, clinical factors and prognosis in cervical and endometrial cancer.

In cervical cancer, ABCF2 expression was higher in stages III and IV than stages I and II tumours but expression was not related to histology or age of the patients. The OS was longer in ABCF2-negative than ABCF2-positive cases (*P*=0.0069). In addition, ABCF2 positivity was shown to be a significant independent prognostic factor by multivariate proportional hazard test. In cervical cancer, squamous cell antigen (SCC) is thought to be a useful marker. Pretreatment SCC levels correlate with cellular differentiation, tumour stage and volume, lymph node status and blood vessel invasion (Reynolds *et al*, 2005). However, there are conflicting reports on the prognostic significance of pretreatment SCC measurements (Scambia *et al*, 1994; Gaarenstroom *et al*, 1995; Duk *et al*, 1996; Ngan *et al*, 1996; Bolger *et al*, 1997; Hong *et al* 1998). In addition, there are no useful markers in cervical adenocarcinoma. In this study, the ABCF2 positivity rate was 61% (25 of 41) in cervical non-squamous cell carcinoma and expression correlated with OS. ATP-binding cassette superfamily F2 may therefore be a useful biomarker in cervical cancer, although a larger study will be needed to confirm this conclusion. In contrast, ABCF2 expression was not related to OS and clinical factors such as age, stage, histologic type, histologic grade and oestrogen receptor status in endometrial cancer. Thus, the role of ABCF2 may differ according to tumour type.

In ovarian cancer, ABCF2 protein expression was related with histologic types (significantly higher in clear-cell type compared with other histologic types) (Tsuda *et al*, 2005a; Nishimura *et al*, 2007). However, in cervical and endometrial cancer, its expression was not related with histologic types. In cervical cancer and clear cell types of ovarian cancer, ABCF2 expression was related with survival, however, in endometrial cancer and other histologic types of ovarian cancer, its expression was not related with survival (Tsuda *et al*, 2005b). The reason remains unclear. The role of ABCF2 protein may differ among histologic types or origin.

In conclusion, ABCF2 expression may be a useful prognostic marker in cervical cancer but its expression is not related to prognosis and clinical factors in endometrial cancer.

## Figures and Tables

**Table 1:** ATP-binding cassette superfamily F2 (ABCF2) expression in cervical cancer

**Parameter**	**ABCF2 expression**	***P*-value**
*Age*		
<Median	50.4% (71/141)	0.065
>Median	61.9% (78/126)	
		
*FIGO stage*		
I+II	50.7% (108/213)	0.001
III+IV	75.9% (41/54)	
		
*Histologic type*		
Squamous	54.9% (124/226)	0.499
Non-squamous	61.0% (25/41)	

**Table 2:** ATP-binding cassette superfamily F2 (ABCF2) expression in endometrial cancer

**Parameter**	**ABCF2 expression**	***P*-value**
*Age*		
<Median	68.6% (35/51)	0.832
>Median	71.2% (37/52)	
		
*FIGO stage*		
I+II	68.1% (47/69)	0.652
III+IV	73.5% (25/34)	
		
*Histologic type*		
Endometrioid	69.6% (64/92)	0.999
Non-endometrioid	72.7% (8/11)	
		
*Histologic grade*		
1+2	68.6% (59/86)	0.773
3	76.5% (13/17)	
		
*Estrogen receptor*		
Positive	71.2% (47/66)	0.384
Negative	58.8% (10/17)	

**Table 3:** Statistical analysis of prognostic factors for OS

**Variable**	**Univariate *P-value***	**Hazard ratio**	**95% CI**
Age	0.0008	1.549	1.199–2.029
Histology	0.17	1.242	0.904–1.651
Clinical stage	<0.0001	2.577	1.993–3.329
ABCF2	0.0063	0.696	0.527–0.904

Age: >median age, histology: non-squamous cell carcinoma.

Clinical stage: III+IV, ABCF2: negative.

Hazard ratio refers to risk of death, with values <1.0 indicating reduced risk.

CI=confidence interval.
